# MiR-33 promotes myocardial fibrosis by inhibiting MMP16 and stimulating p38 MAPK signaling

**DOI:** 10.18632/oncotarget.25173

**Published:** 2018-04-24

**Authors:** Zhen Chen, Hua-Sheng Ding, Xin Guo, Jing-Jing Shen, Di Fan, Yan Huang, Cong-Xin Huang

**Affiliations:** ^1^ Department of Cardiology, Renmin Hospital of Wuhan University, Wuhan University, Wuhan 430060, PR China; ^2^ Institute of Cardiovascular Diseases, Wuhan University, Wuhan 430060, PR China; ^3^ Hubei Key Laboratory of Cardiology, Wuhan 430060, PR China

**Keywords:** myocardial fibrosis, cardiac fibroblasts, miRNA-33, matrix metalloproteinase 16, p38 MAPK signaling pathway

## Abstract

Myocardial fibrosis occurs in the late stages of many cardiovascular diseases, and appears to be stimulated by various microRNAs (miRNAs). We previously found that miR-33 may stimulate cardiac remodeling. Here, we examined the involvement of miR-33 in myocardial fibrosis. Proximal left coronary descending artery occlusion was performed in rat, and antagomiR-33a was injected. Primary cardiac fibroblasts were cultured and transfected with miR-33a mimics and inhibitors. miR-33a levels were increased in the rat after surgery, and collagen deposition and heart fibrosis were observed *in vivo*. Inhibition of miR-33a suppressed fibroblast proliferation, reduced the mRNA and protein levels of collagen-related markers *in vitro* and *in vivo*, and rescued the histological damage *in vivo*. A dual-luciferase reporter system showed that matrix metalloproteinase 16 (MMP16) gene was the direct target of MiR-33a. These results suggest that miR-33 promoted myocardial fibrosis by inhibiting MMP16 and stimulating p38 mitogen-activated protein kinase (p38 MAPK) signaling pathway. MiR-33 may act as a novel therapeutic target for treating myocardial fibrosis.

## INTRODUCTION

Myocardial fibrosis (MF) occurs in the late stages of many cardiovascular diseases, and it targets cardiac fibroblasts (CFs). Under normal conditions, CFs represent at least 60% of the total cells in the heart. Within a damaged heart, CFs are rapidly activated by microenvironment signals to remove dead cardiomyocytes, generate collagen-enriched ECM, and repair the damaged areas [1]. In this process, cardiomyocytes showed negligible regenerative abilities, while CFs invaded and destroyed the architecture of the heart. Because CF activation promotes MF, CFs are a prospective therapeutic target to diminish MF [2].

MicroRNA (miR) is small non-coding RNA that inhibits gene expression at a post-transcriptional level by binding to the complementary sequences in the 3′ untranslated regions (3-UTRs) of their target genes [2, 3]. MicroRNAs can have anti-fibrotic functions, pro-fibrotic functions, or serve as MF biomarkers [2, 4]. MiR-33a, a highly conserved microRNA ubiquitously expressed in CFs [5], is a potential therapeutic target for cardiovascular diseases because of its correlation with high-density lipoprotein cholesterol (HDL-C) [6]. Anti-miR33 therapy inhibits mitochondrial respiration and ATP production, which in conjunction with increased ABCA1 expression, works to promote macrophage cholesterol efflux and reduce atherosclerosis [5]. We assessed miR-33’s function in heart fibrosis.

## RESULTS

### MiR-33a was increased *in vitro* and *in vivo*

**Figure 1:**
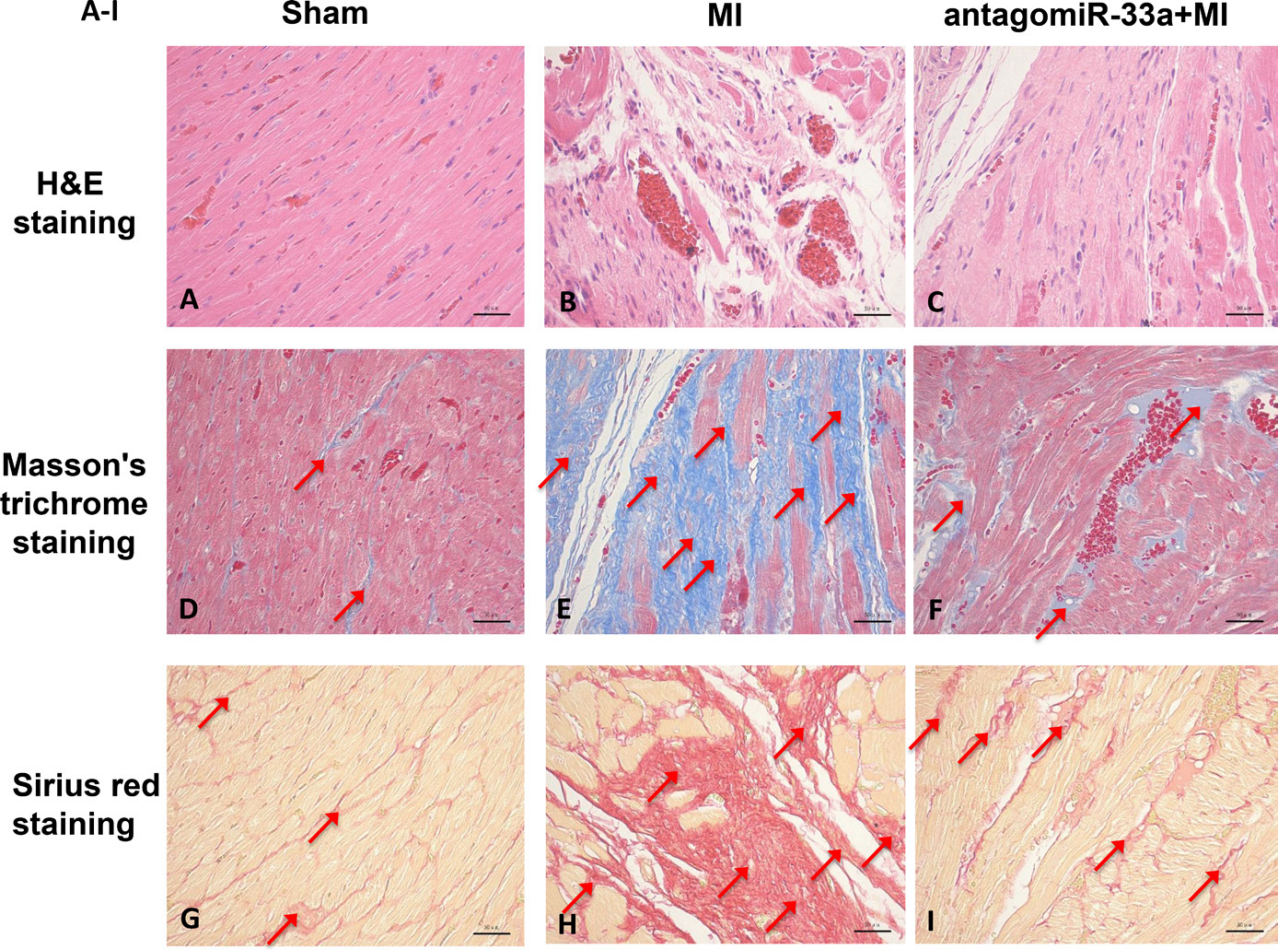

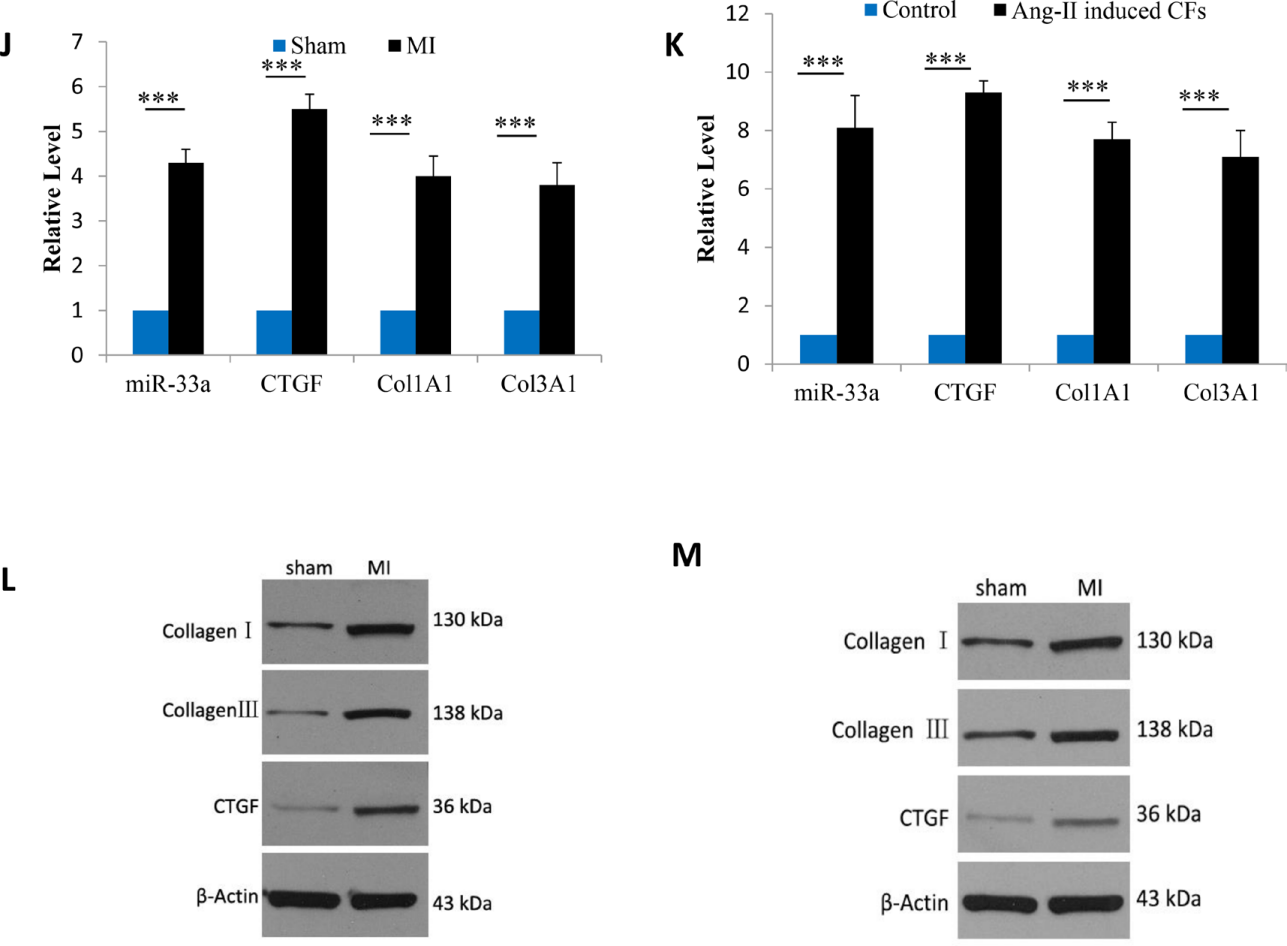
MiR-33a was increased *in vitro* and *in vivo*; myocardial fibrosis observed *in vivo* Representative micrographs of H&E-stained (**A–C**, top row), Masson’s trichrome-stained (**D–F**, middle row) and Sirius red-stained (**G–I**, bottom row) heart tissue sections from sham operated- (left column), myocardial infarction (MI) (middle column), and antagomiR-33a injected- (right column) animals. (**J**) The mRNA levels of miR-33a, CTGF, Col1A1, and Col3A1 in heart tissues were examined by qPCR between MI and sham operated rats. (**K**) The mRNA levels of miR-33a, CTGF, Col1A1, and Col3A1 in CFs were examined by qPCR between Ang-II-induced CFs and normal CFs. (**L**) The protein levels of CTGF, Col1A1, and Col3A1 in heart tissues were examined by Western blot between MI and sham operated rats. (**M**) The protein levels of CTGF, Col1A1, and Col3A1 in CFs were examined by Western blot between Ang-II-induced CFs and normal CFs. Arrows indicate collagen deposition in (D–I). Data are expressed as mean ± SD (*n* = 3). ^*^p < 0.05, ^**^p < 0.01, ^***^p < 0.001, no significance is indicated as “NS”. Scale bars: 50 µm.

The Masson’s trichrome staining and Sirius red staining of heart tissue sections both revealed typical collagen deposition at 28 days post-surgery (Figure 1A, 1B, 1D, 1E, 1G, and 1H). We also found that CTGF, Col1A1, and Col3A1 levels increased in heart tissues according to qPCR (Figure 1J) and Western blot (Figure 1L). The same techniques revealed that Ang-II exposure in CFs resulted in more CTGF, Col1A1, and Col1A3 than in non-treated cells (Figure 1K, 1M). In addition, miR-33a levels increased in the heart tissues of MI (Figure 1J) and Ang-II induced CFs (Figure 1K).

### Inhibition of miR-33a improved echocardiographic function *in vivo*

**Figure 2:**
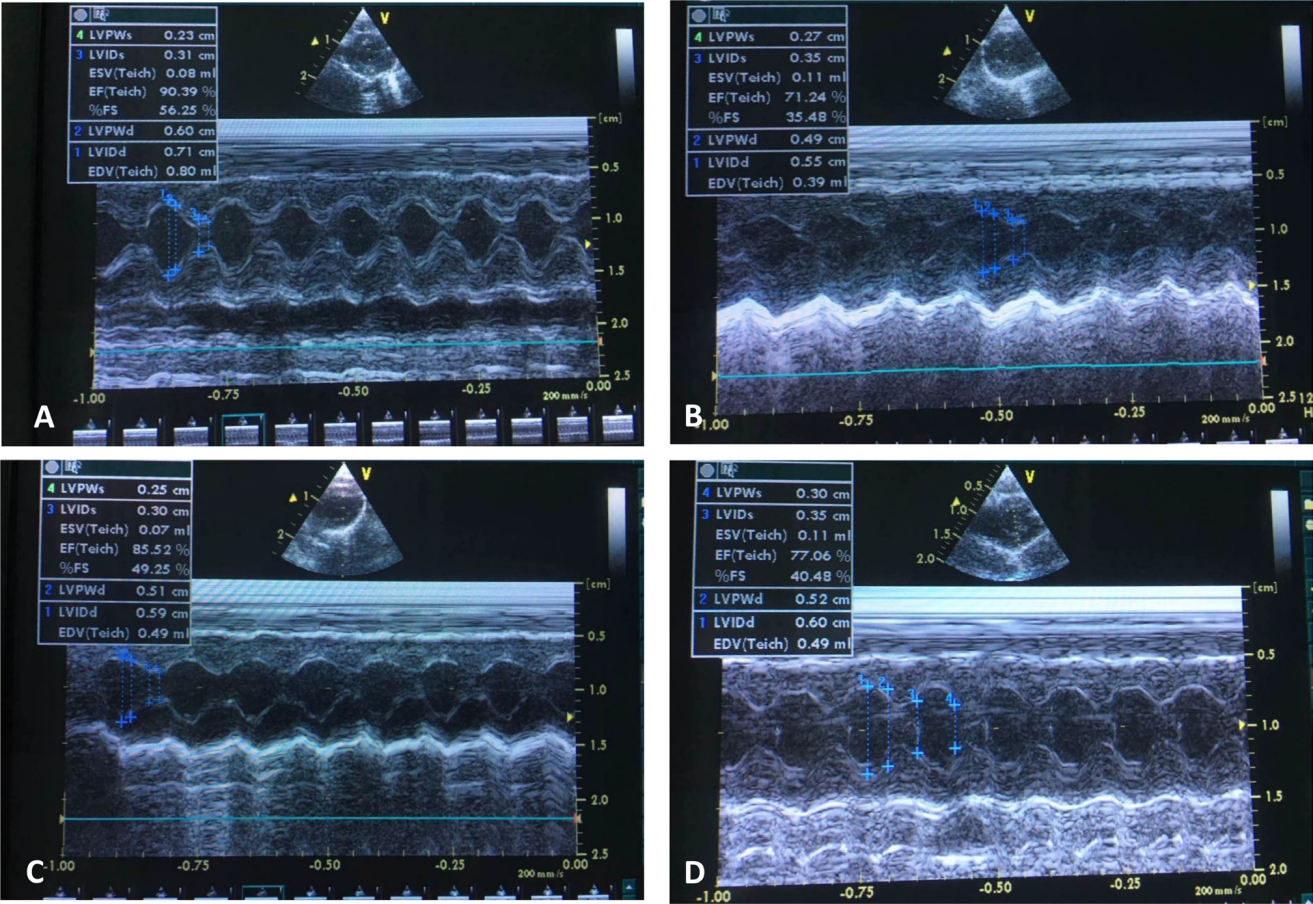

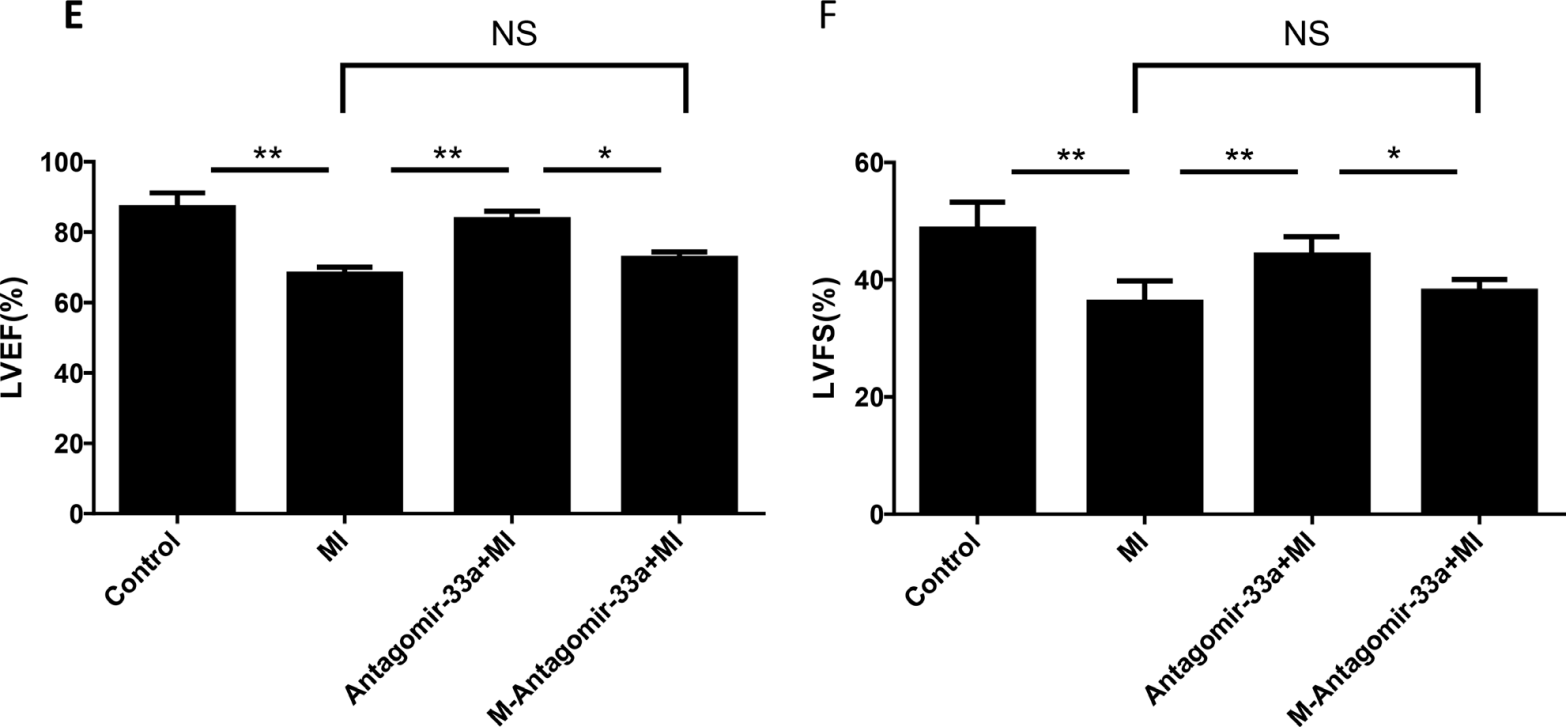
Inhibition of miR-33a improved echocardiographic function *in vivo* Echocardiography was performed at the end of the study (*n* = 8). Representative M-mode images of sham operated- (**A**), myocardial infarction (MI)- (**B**), antagomiR-33a injected- (**C**), and mismatch antagomiR-33a (M-antagomiR-33a) (**D**) animals. LVEF (**E**) and LVFS (**F**) were obtained. Data are expressed as mean ± SD (*n* = 3). ^*^p < 0.05, ^**^p < 0.01, ^***^p < 0.001, no significance is indicated as “NS”.

We evaluated miR-33a inhibition on LV remodeling and function via echocardiography (Figure 2A–2D). After permanent LAD occlusion, antagomiR-33a injection increased LVEF and LVFS levels, but mismatch antagomiR-33a (M-antagomiR-33a) injection did not (Figure 2E, 2F).

### Inhibition of miR-33a suppressed fibrogenesis *in vitro* and *in vivo*

**Figure 3:**
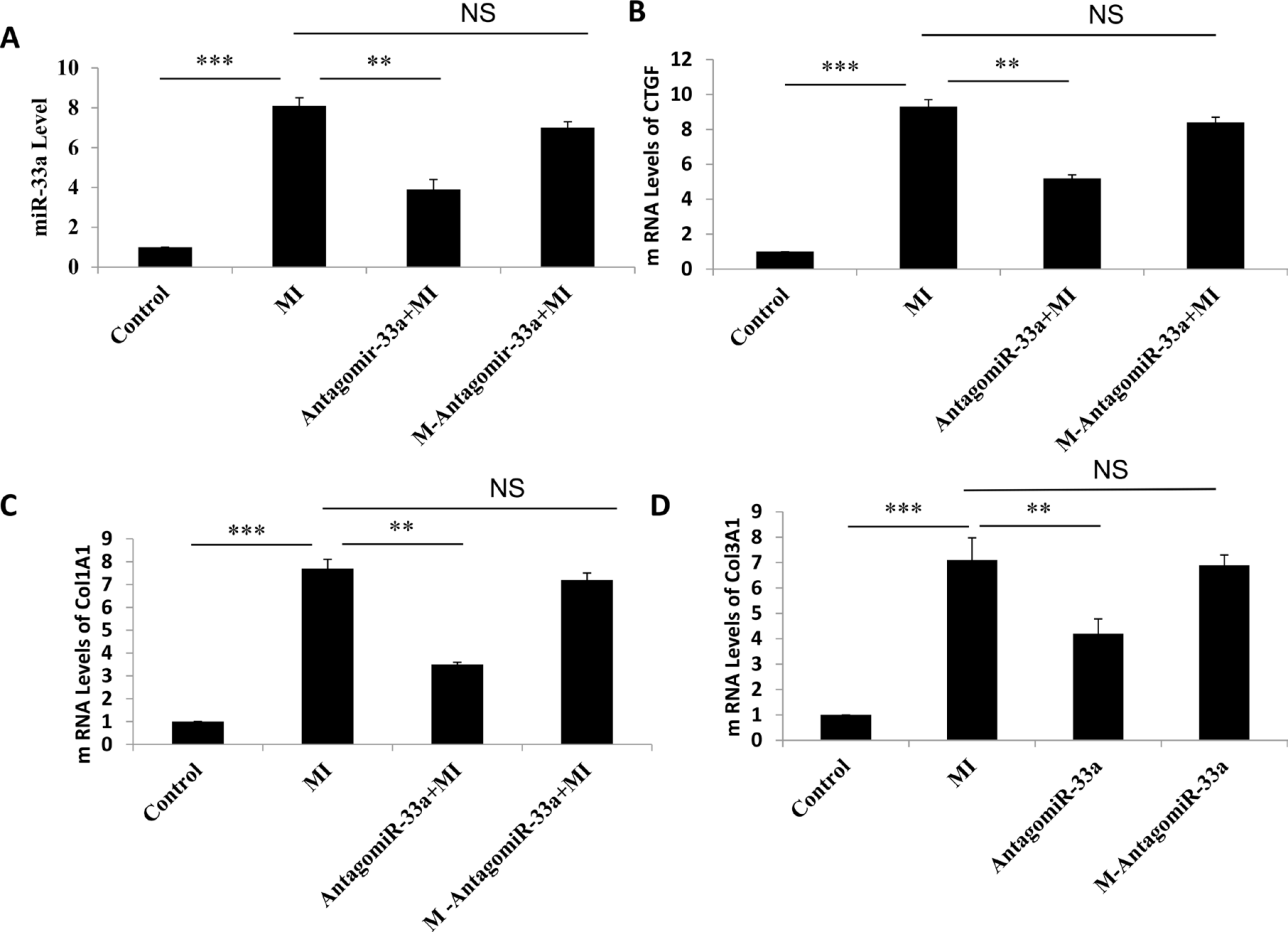

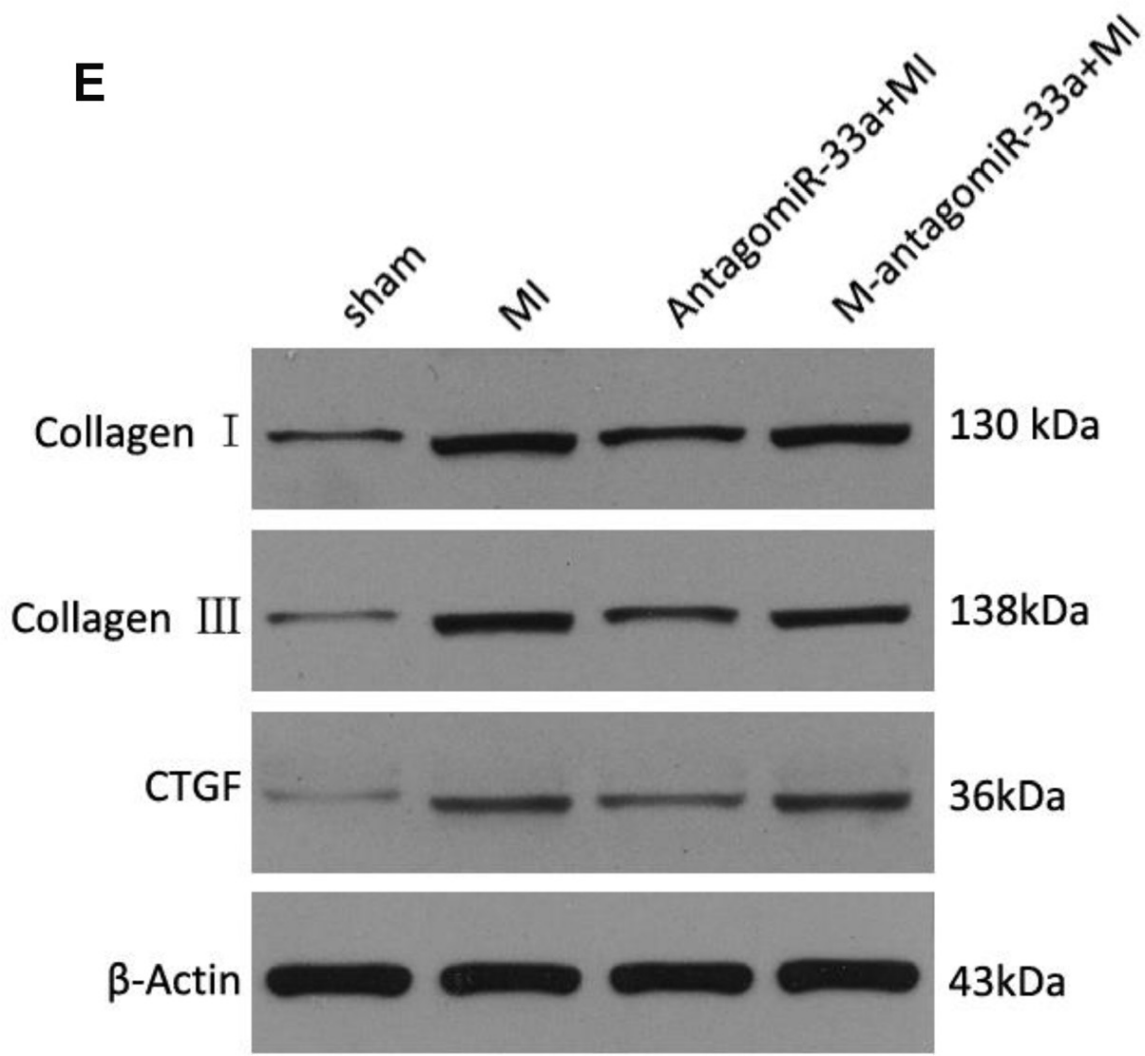
Inhibition of miR-33a suppressed fibrogenesis *in vivo* The mRNA levels of miR-33a (**A**), CTGF (**B**), Col1A1 (**C**), and Col3A1 (**D**) in heart tissues were examined by qPCR among sham rats (Control), MI rats (MI), antagomiR-33a injected MI rats (antagomiR-33a+MI), and mismatch antagomiR-33a injected MI rats (M-antagomiR-33a+MI). (**E**) The protein levels of CTGF, Col1A1, and Col3A1 in heart tissues were examined by Western blot among sham, MI, antagomiR-33a+MI, and M-antagomiR-33a+MI. Data are expressed as mean ± SD (*n* = 3). ^*^p < 0.05, ^**^p < 0.01, ^***^p < 0.001, no significance is indicated as “NS”.

To investigate if miR-33a inhibition inhibited fibrogenesis, we injected antagomiR-33a to animals through the tail vein. AntagomiR-33a inhibition reduced miR-33a levels in heart tissues (Figure 3A), rescued the histological damage (Figure 1C, 1F, 1I), and reduced the mRNA and protein levels of CTGF, Col1A1 and Col3A1 (Figure 3B–3E). However, mismatch antagomiR-33a (M-antagomiR-33a) failed to show these effects (Figure 3B–3E).

**Figure 4:**
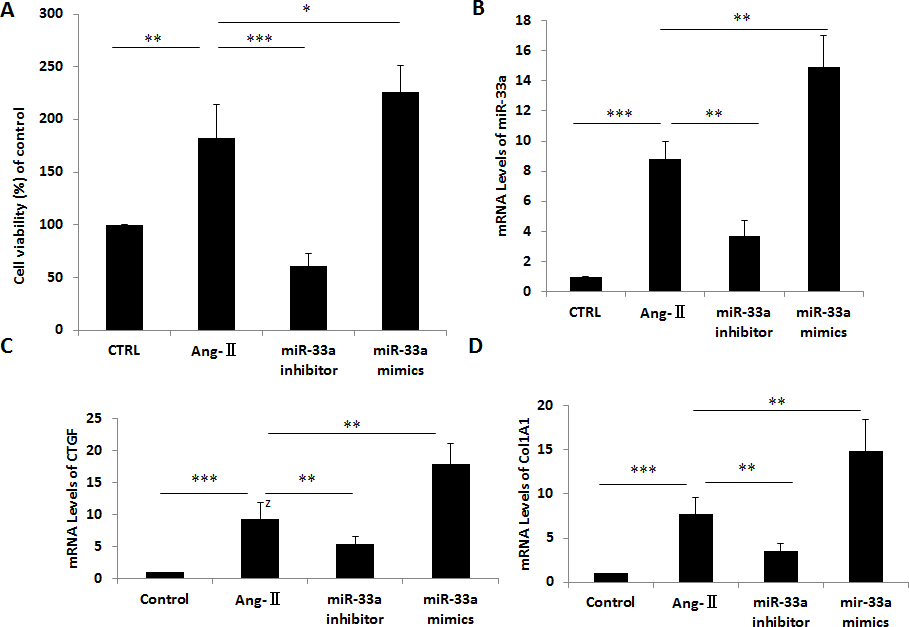

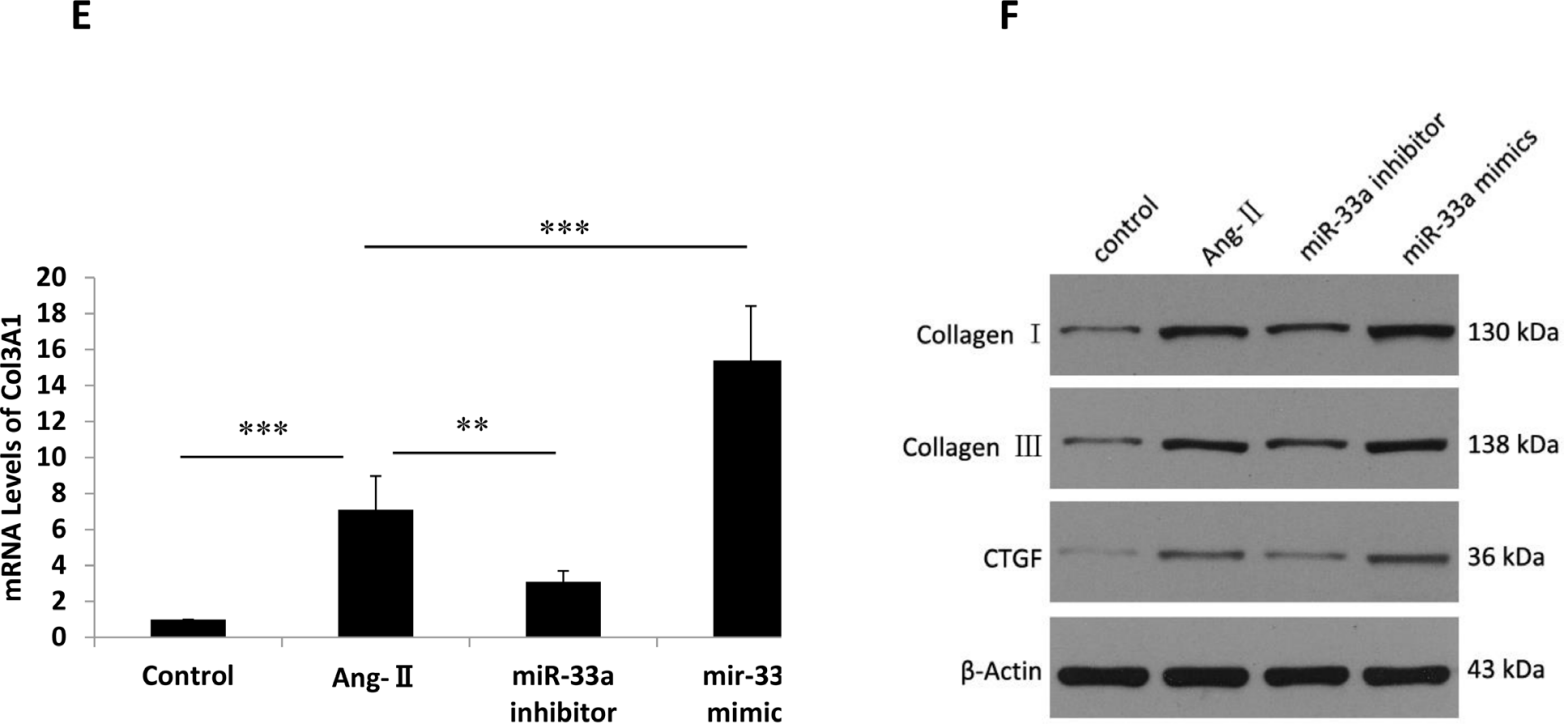
Inhibition of miR-33a suppressed fibrogenesis *in vitro* (**A**) Cell viability was analyzed by CCK8 in control cells (CTRL), Ang-II-induced CFs (Ang-II), miR-33a inhibitor treated CFs (miR-33a inhibitor), and miR-33a mimics treated CFs (miR-33a mimics). (**B–E**) The mRNA levels of miR-33a (B), CTGF (C), Col1A1 (D), and Col3A1 (E) were examined by qPCR in control cells (CTRL), Ang-II-induced CFs (Ang-II), miR-33a inhibitor treated CFs (miR-33a inhibitor), and miR-33a mimics treated CFs (miR-33a mimics). (**F**) The protein levels of CTGF, Col1A1, and Col3A1 were examined by Western blot in control cells (CTRL), Ang-II induced CFs (Ang-II), miR-33a inhibitor treated CFs (miR-33a inhibitor), and miR-33a mimics treated CFs (miR-33a mimics). Data are expressed as mean ± SD (*n* = 3). ^*^p < 0.05, ^**^p < 0.01, ^***^p < 0.001, no significance is indicated as “NS”.

MiR-33a inhibition in CFs by miR-33a inhibitor reduced proliferation rate (Figure 4A) and lowered mRNA levels of miR-33a, CTGF, Col1A1, and Col3A1 (Figure 4B–4E) compared with non-treated cells by qPCR. Inhibition of miR-33a in CFs also reduced protein levels of CTGF, Col1A1, and Col3A1 by Western blot (Figure 4F). Overexpression of miR-33a in CFs by miR-33a mimics increased RNA and protein levels of CTGF, Col1A1, and Col3A1 by qPCR and Western blot (Figure 4C–4F).

### MiR-33a mediated fibrogenesis via the direct target gene, MMP16

**Figure 5:**
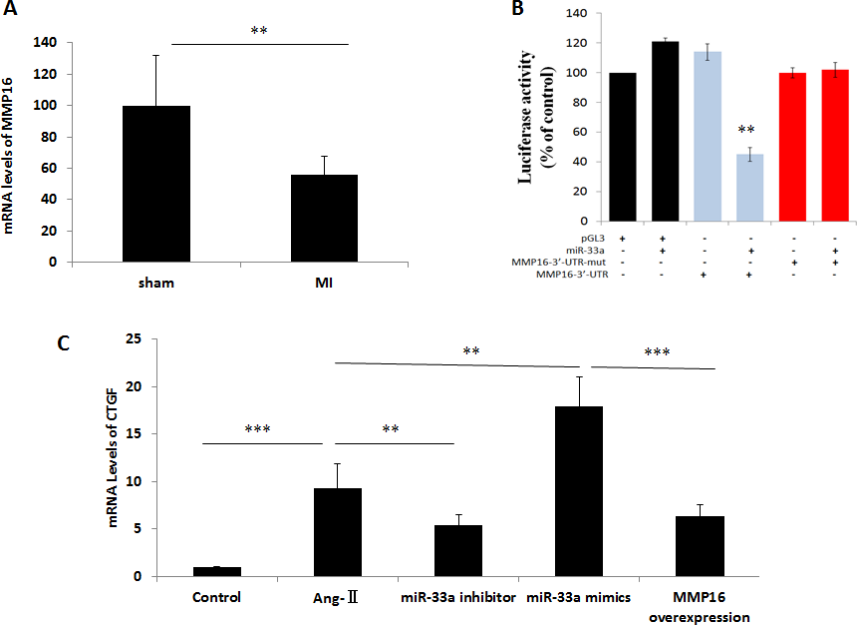

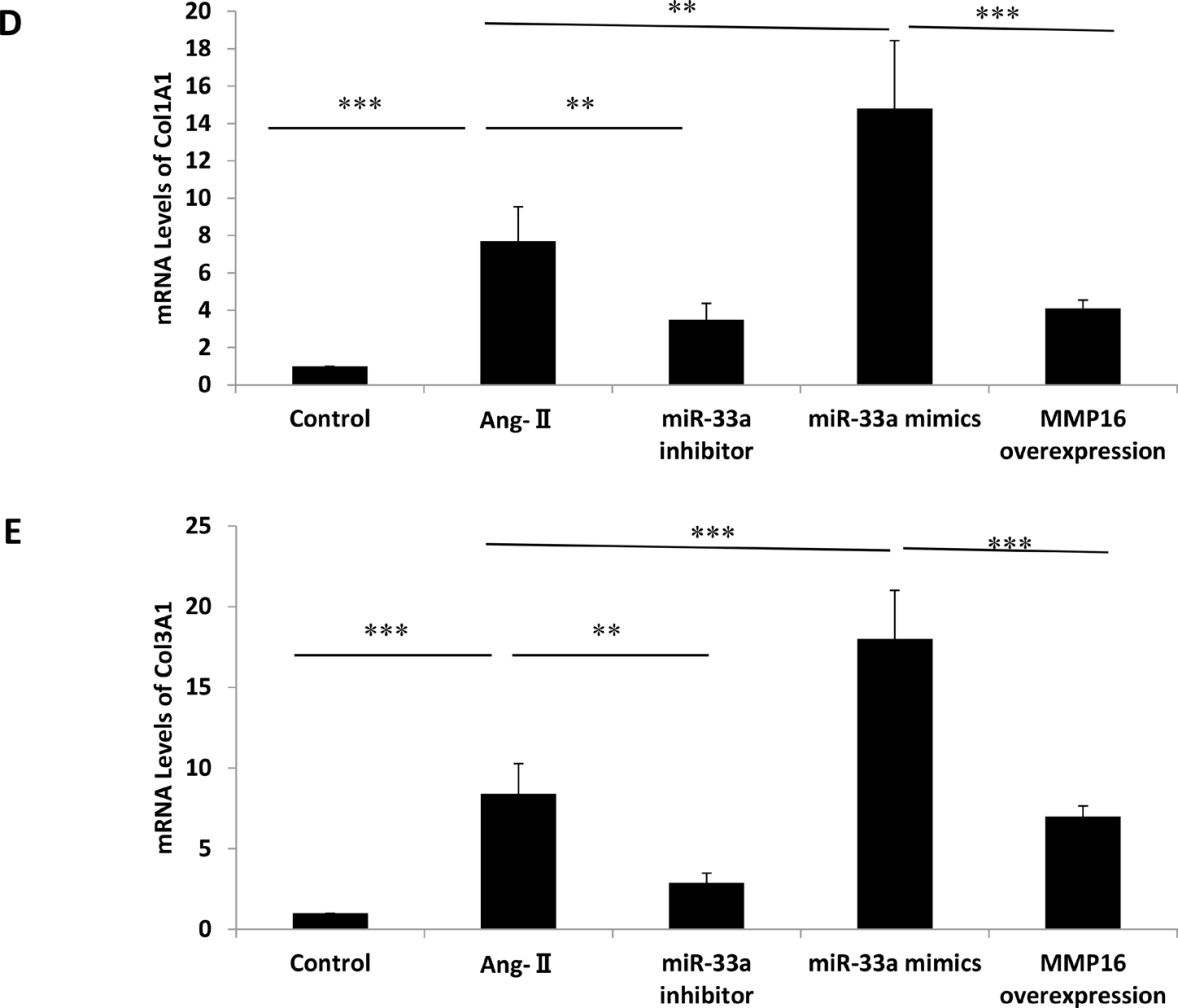
MiR-33a promoted fibrogenesis via its direct target gene, MMP16 (**A**) The mRNA levels of MMP16 in heart tissues were examined by qPCR between sham rats, and MI rats (MI). (**B**) Validation of MMP16 as a miR-33 target gene by luciferase-activity assay. Introduction of mutations in the 3′-UTR of MMP16 blocked inhibition of MMP16 translation. (**C–E**) The mRNA levels of CTGF (C), Col1A1 (D), and Col3A1 (E) were examined by qPCR in control cells (CTRL), Ang-II-induced CFs (Ang-II), miR-33a inhibitor treated CFs (miR-33a inhibitor), and MMP16 over-expressed CFs (MMP16 overexpression). Data are expressed as mean ± SD (*n* = 3). ^*^p < 0.05, ^**^p < 0.01, ^***^p < 0.001, no significance is indicated as “NS”.

To confirm potential miR-33a target genes, we used the online prediction programs TargetScan (www.targetscan.org) and miRBase (www.microrna.org). Among all these potential genes, MMP16 was considered to promote the fibrosis process [7, 8]. MMP16 levels in heart tissues reduced after surgery (Figure 5A). To verify whether miR-33a directly binds to the 3′-UTR of rat MMP16 mRNA in the post-transcriptional level, we cloned the wild-type and mutated 3′- UTR sequence of MMP16 into a dual-luciferase reporter vector. Wild-type 3′-UTR of MMP16 (MMP16-3′-UTR) reduced the luciferase activity in the presence of miR-33a, whereas miR-33a did not affect the luciferase activity of the mutated 3′-UTR of MMP16 (MMP16-3′-UTR-mut) (Figure 5B). MMP16 overexpression inhibited miR-33a mimics-induced increases of CTGF, Col1A1, and Col3A1 in CFs (Figure 5C–5E). However, similar effects were not observed using empty control (data not shown).

### MiR-33a controlled fibrogenesis through p38 MAPK but not TGF-β/Smad pathway

**Figure 6:**
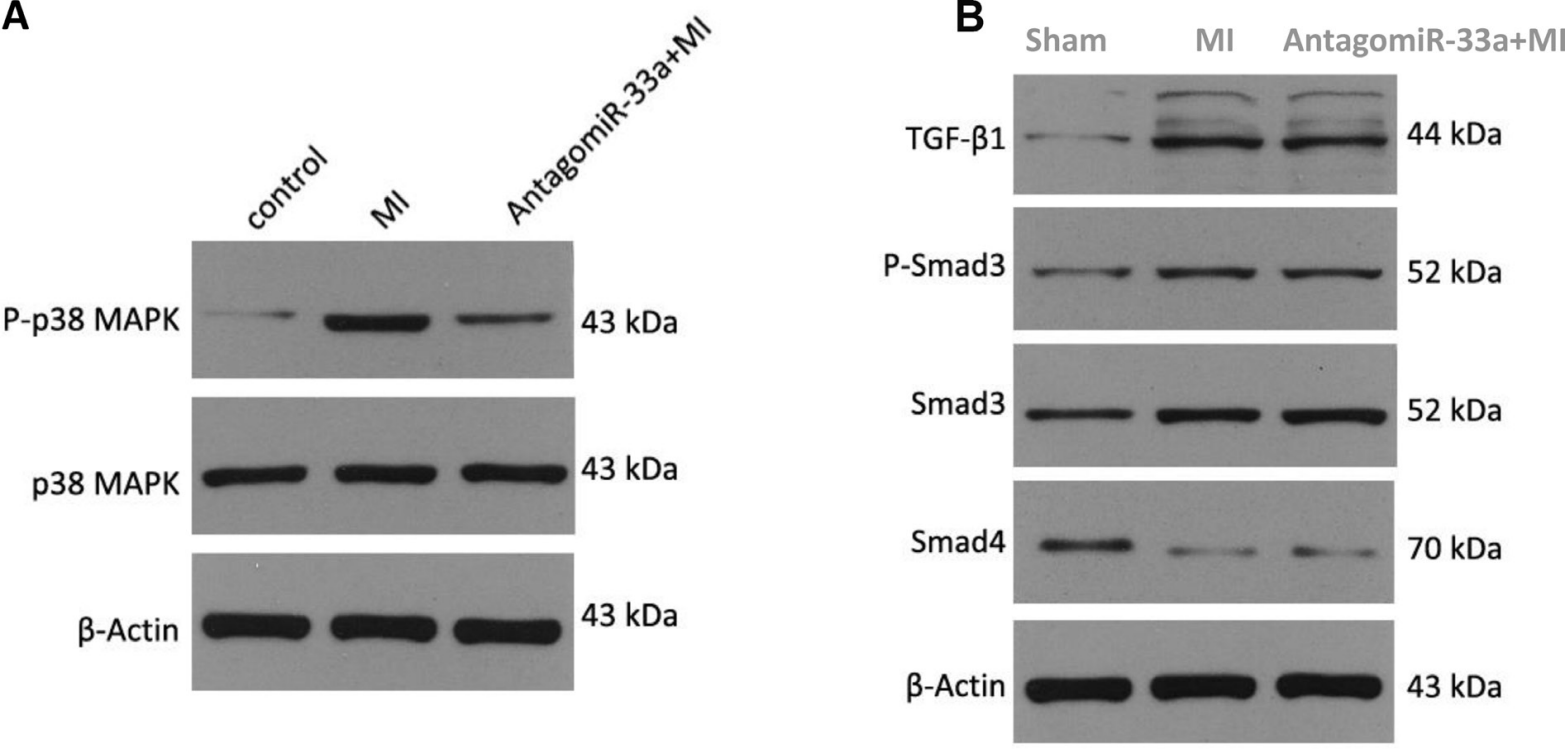
MiR-33a stimulates fibrogenesis through p38 MAPK but not TGF-β/Smad pathway (**A**) The protein levels of phospho-p38 MAPK, total p38 MAPK in heart tissues were examined by Western blot among sham rats (Control), MI rats (MI), antagomiR-33a injected MI rats (antagomiR-33a+MI). (**B**) The protein levels of TGF-β1, phospho-Smad3, Smad3, Smad4 in heart tissues were examined by Western blot among sham rats (Control), MI rats (MI), antagomiR-33a injected MI rats (antagomiR-33a+MI). Data are expressed as mean ± SD (*n* = 3). ^*^p< 0.05, ^**^p < 0.01, ^***^p < 0.001, no significance is indicated as “NS”.

Myocardial fibrosis can be promoted by the p38 MAPK [9] and TGF-β/Smad signaling pathways [10]. We examined whether miR-33a used either of those signaling pathways to stimulate myocardial fibrogenesis. We found that surgery in rat increased phospho-p38 MAPK levels at 28 days post-surgery, and antagomiR-33a injection inhibited this pathway (Figure 6A). While Smad3 and TGF-β1 protein levels in the TGF-β/Smad signaling pathway were also increased in the animals at 28 days post-surgery, they were not reduced by antagomiR-33a injection (Figure 6B). Smad3 phosphorylation (Ser423/425) was also not suppressed by antagomiR-33a injection (Figure 6B). In contrast, Smad4 protein decreased in the animals at 28 days post-surgery, and were not increased by antagomiR-33a injection (Figure 6B).

## DISCUSSION

A series of miRNAs are strongly associated with the pathogenesis of myocardial fibrosis, including miR-33a [11–17]. MiR-33a and miR-33b are conserved miRNA located in intron 16 of SREBP-2 and SREBP-1, respectively [18]. They are the most abundant miRNAs in lipoprotein particles [19]. Cardiac fibrosis can be ameliorated in miR-33 knockout hearts, and cardiac fibroblasts (CFs) are mainly responsible for miR-33 expression in the heart [5]. Long-term therapeutic silencing of miR-33 increases circulating triglyceride levels and hepatic lipid accumulation in mice [20], and genetic permanent miR-33 inhibition may cause cardiac dysfunction [5]. Considering these harmful side effects, we adopted the tentative silencing of miR-33 approach by antagomiR-33a. No prominent side effects were observed. In the present study, miR-33a was increased in rat hearts after myocardial infarction. Additionally, miR-33a inhibition suppressed cardiac fibrosis *in vivo*, as well as collagen formation and fibrogenesis *in vitro*, by targeting MMP16.

We used online prediction programs to predict all target genes of miR-33, and MMP16 was screened for further research because MMPs are a group of proteolytic enzymes that are responsible for the maintenance of the ECM [21]. MMP16 expression is decreased in dupuytren’s disease patients, which is a typical fibrosis disease [8]. Decreased MMP16 levels have also been verified in Ang II-induced miRNAs in cardiac fibroblasts [22]. MMP16 protein activates MMP2 protein which in turn degrades type III collagen [8]. We demonstrated that MMP16 levels decreased in the heart tissues, and MMP16 overexpression inhibited miR-33a mimics-induced increases of CTGF, Col1A1, and Col3A1 in CFs. Our dual-luciferase reporter assay confirmed that MMP16 was a direct target gene of miR-33a.

TGF-β/Smad signaling stimulates myocardial fibrogenesis [10]. Western blot analysis revealed that miR-33a/MMP16 did not promote fibrogenesis through TGF-β/Smad signaling. Previous studies demonstrated that TGF-β/Smad signaling interacted with MAPK signaling in fibrosis disease [23]. We then examined whether miR-33a controlled fibrogenesis through MAPK signaling by western blot, and found that phosphorylated p38 was reduced in the antagomiR-33a injection-treated heart tissues more than the un-treated heart tissues.

We performed a comprehensive study of miR-33 expression in a rat cardiac fibrosis model *in vivo*, Ang-II-treated primary cardiac fibroblasts *in vitro*, and miR-33 target genes. We found miR-33a was increased in the rat after surgery, and collagen deposition and heart fibrosis were observed *in vivo* simultaneously. We also found that miR-33a inhibition inhibited CF proliferation rate, reduced the mRNA and protein levels of collagen-related markers *in vitro* and *in vivo*, and rescued the histological damage *in vivo*. We obtained the opposite results when miR-33a was over-expressed in cultured CFs by miR-33a mimics. Furthermore, we found that MMP16 gene was the direct target for miR-33a in CFs, and that miR-33a promoted cardiac fibrosis via the p38 MAPK signaling pathway. Our results indicate miR-33a could be a novel therapeutic target for myocardial fibrosis.

## MATERIALS AND METHODS

### Animal welfare and ethical approval

Eight-week-old Sprague-Dawley (SD) rats were housed according to United States National Institutes of Health (NIH) guidelines and the local committee for the care and use of laboratory animals. The rats were housed three rats per cage prior to surgery, and one rat per cage after surgery. All surgical procedures and animal experiments were approved by the welfare committee for animal use and care, and performed according to the Guide for the Care and Use of Laboratory Animals published by the NIH. Every effort was made to minimize the number of animals used, as well as their suffering.

### Animal model of myocardial fibrosis and miR-33a treatment

SD rats were anesthetized with 3% sodium pent barbiturate (50 mg/kg), intubated with a polyethylene catheter, and room air was continuously provided by a rodent ventilator. The chest was opened by left thoracotomy between the third and the fourth intercostal space, and the pericardium was opened and removed. The proximal left coronary descending artery (LAD) was permanently encircled and ligated with an intramural stitch, then the chest was closed. High mortality occurred during induction procedures, and rats that died within 24h after surgery were excluded for further analysis. Sham-operated rats were subjected to the same procedure, but without ligation.

The cholesterol-conjugated miR-33a antisense (antagomiR-33a) and mismatch antagomiR-33a (M-antagomiR-33a) were purchased from RiboBio Co., Ltd. (Guangzhou, China) [7, 8, 11, 24]. The antagomiR-33a and M-antagomiR-33a were injected through the tail vein at 40 mg/kg for seven consecutive days after surgery [7, 24]. These constructs were chemically modified and conjugated with cholesterol moiety for *in vivo* applications with long-lasting stability and enhanced target specificity and affinity [11].

### Echocardiography

Echocardiographic analyses were performed 28 days after operation. Rats were anesthetized with 50 mg/kg ketamine and 10 mg/kg xylazine. The chest was shaved, and the rats were placed supine. Echocardiograms were performed with a commercially available echocardiography system equipped with 7.5- MHz phased-array transducer (Hewlett-Packard). The transducer was positioned on the left anterior side of the chest after the precordium was shaved. We evaluated the left ventricle (LV) dimensions in parasternal short-axis view during systole or diastole. We measured left ventricular fractional shortening (LVFS), left ventricular ejection fraction (LVEF) from the LV M-mode tracing with a sweep speed of 50 mm/s at the mid-papillary muscle level.

### Sample collection and histological analysis

The rats were sacrificed 28 days post-surgery. Some hearts were fixed with 4% paraformaldehyde for histological and immunohistochemical analysis, while some hearts were snap frozen with dry ice powder and stored in liquid nitrogen tank until use for qPCR and Western blot analysis. The hearts were dehydrated in ethanol, embedded in paraffin, sectioned at 4 μm thickness, and stained with hemotoxylin/eosin (H&E), Masson’s trichrome, and Sirius red.

### Primary cardiac fibroblasts culture

Primary CFs were isolated and purified according to previously described methods [11]. Newly dissected hearts were collected from 1 to 3-day-old SD rats, and immediately rinsed in cold PBS several times until the blood was cleared. The hearts were minced, and then digested with 0.25% trypsin and 0.02% EDTA (Sigma, USA) at 37° C for 1h. The digestion was stopped by adding Dulbecco’s Modified Eagle’s Medium (DMEM) (Hyclone, Logan, UT, USA) medium with 10% fetal bovine serum (FBS) (Hyclone), 100 U/ml penicillin, and 100 ug/ml streptomycin. The pellets were gently re-suspended with 10% FBS/DMEM, and then cultured in plates for 2 h at 37° C in a CO_2_ incubator. The non-adherent cardiomyocytes were gently changed off with 10% FBS/DMEM, and the attached cells were cultured. At 70–80% confluency, cells were passaged, and cells at passage 2–6 were used for *in vitro* studies.

### CFs transfection and ang-II treatment

CFs at 70–80% confluency were replaced and incubated with serum-free medium for 24h before transfection. The cells were then transfected with miR-33a mimic, miR-33a inhibitor (anti-miR-33a), or the negative control (NC) (100 nM each final concentration, Invitrogen, USA) by Lipofectamin 2000 according to the manufacturer’s protocols (Invitrogen, Carlsbad, CA). After transfection, CFs were washed twice with PBS, replaced with fresh 10% FBS/DMEM medium, and treated with 10^–7^ mol/L Ang-II for 24 h [24].

### Overexpression of MMP16 in CFs

CFs were seeded at 1 × 10^5^/well in 6-well plates, then transfected with MMP16 overexpression plasmid (pEGFP-N1-MMP16) or empty plasmid (pEGFP-N1) (GenePharma, Shanghai, China) by Lipofectamin 2000 (Invitrogen, Carlsbad, CA) according to the manufacturer’s instructions.

### Cell viability assay

CF cell viability was measured by CCK8 assay (cell counting kit-8, Dojindo Molecular Technologies, Tokyo, Japan) according to the manufacturer’s instructions. CFs were seeded at 6 × 10^3^ cells per well in 96-well plates and treated as described above. CCK-8 solution (10 μl/well) was added to the wells, and the plate was incubated for 2 h at 37° C. The absorbance of each well was determined at 450 nm using a microplate reader, and the growth curve was calculated.

### Reverse transcription real-time polymerase chain reaction (RT-qPCR)

CFs were treated and collected as described above. Total RNA was extracted from CFs and heart tissues by Trizol reagent (TaKaRa, Tokyo, Japan). First strand complementary DNA (cDNA) was synthesized using the Reverse Transcription Kit (TaKaRa, Tokyo, Japan) according to manufacturer’s instructions. For quantitative PCR (qPCR), PCR amplifications were quantified using the SYBR Green PCR Master Mix (Applied Biosystems) and normalized to GAPDH gene expression. MiR-33a quantification was performed with a stem-loop real-time PCR miRNA kit (Ribobio, Guangzhou, China).

The sequences of the q-PCR primers are described below: miR-33a (forward: 5′-GATCCTCAGTGCATTGTAGTTGC-3′; reverse: 5′-CTCTGTCTCT CGTCTTGTTGGTAT-3′), Col1A1 (forward: 5′-TCCTGACGCATGGCCAAGAA-3′; reverse: 5′-CATAGCACGCCATCGCACAC-3′), Col3A1 (forward: 5′-TGGACAGATGCTGGTGCTGAG -3′; reverse: 5′-GAAGGCCAGCTGTACATCAAGGA-3′), CTGF (forward: 5′-GCAGCTAGAGAAGCAGAGC-3′; reverse: 5′-GGTGCAGCCAGAAAGCTC-3′), MMP16 (forward: 5′-AATCTCCTCAGGGAGCATTTGTA-3′; reverse: 5′-TCCAGGTTCTACC TTGAGTATCTG-3′), U6 (forward: 5′-ATTGGAACGATACAGAGAAGATT-3′; reverse: 5′-GGAACGCTTCACGAATTTG-3′), and GAPDH (forward: 5′-GAC ATG CCG CCT GGA GAA AC-3′; reverse: 5′-AGC CCA GGA TGC CCT TTA GT-3′).

### Luciferase assays

The wild-type 3′-UTR of rat MMP16 containing the putative binding site for miR-33a was amplified by PCR. The mutated 3′-UTR of rat MMP16 was generated using a QuikChange II XL site-directed mutagenesis kit (Stratagene, San Diego, CA, USA), and the constructs were verified by sequencing. Wild-type and mutated 3′-UTR regions were then sub-cloned into the pGL3 vector immediately downstream of the luciferase gene stop codon (Promega, USA). HEK293 cells were co-transfected with 100 ng of the plasmid constructs, MMP16-3′UTR, or MMP16-3′UTR-mut, and infected with plasmids expressing miR-33a or pGL3. After 48 hours, luciferase activities were measured with a dual-luciferase reporter assay kit (Promega) according to the manufacturer’s instructions.

### Western blot

Proteins were extracted with RIPA lysis buffer (Beyotime, Jiangsu, China) from homogenized frozen heart tissues, as well as cells. Protein concentrations were measured by Bradford assay. Equal protein amounts were loaded into SDS-PAGE gels and separated by electrophoresis. Primary antibodies were used to detect CTGF (1:1000, Sigma-Aldrich, USA), Collagen I (1:5000, Sigma-Aldrich, USA), Collagen III (1:5000, Sigma-Aldrich, USA), and actin (1:10000, Sigma-Aldrich, USA). Western blot bands were scanned and quantified by Image J software.

### Statistical analysis

All experiments were conducted at least in triplicate, and representative data are expressed as the mean ± SD. The comparisons were evaluated by one-way analysis of variance and for significant relationships, post-hoc multiple comparisons between means were evaluated with the Turkey test. All statistical analyses were performed using SPSS statistics software, and *p* < 0.05 was considered significant.
